# Short-term and long-term oncological outcomes of chemoradiotherapy for rectal cancer patients with or without oxaliplatin: a propensity score-matched retrospective analysis

**DOI:** 10.1186/s13014-024-02562-y

**Published:** 2024-12-03

**Authors:** Amirali Azimi, Fatemeh Sadat Tabatabaei, Kasra Kolahdouzan, Hamideh Rashidian, Forouzan Nourbakhsh, Maryam Abedini Parizi, Nima Mousavi Darzikolaee, Reyhaneh Bayani, Samaneh Salarvand, Azadeh Sharifian, Farzaneh Bagheri, Saeed Rezaei, Naeim Nabian, Reza Nazari, Negin Mohammadi, Mohammad Babaei, Marzieh Lashkari, Farshid Farhan, Mahdi Aghili, Felipe Couñago, Maria Antonietta Gambacorta, Reza Ghalehtaki

**Affiliations:** 1 Radiation Oncology Research Center, Cancer Research Institute, Tehran University of Medical Sciences, Tehran, Iran; 2 Department of Radiation Oncology, Cancer Institute, School of Medicine, IKHC, Tehran University of Medical Sciences, Tehran, Iran; 3 Cancer Research Center, Cancer Research Institute, Tehran University of Medical Sciences, Tehran, Iran; 4 Department of Anatomical and Clinical Pathology, School of Medicine, IKHC, Tehran University of Medical Sciences, Tehran, Iran; 5 Department of Radiation Oncology, Hospital Universitario Vithas Madrid La Milagrosa, GenesisCare, Madrid, Spain; 6 Department of Radiology, Radiation Oncology and Hematology, Catholic University of the Sacred Heart, Agostino Gemelli University Hospital Foundation IRCCS, 00168 Roma, Italy; 7 Radiation Oncology Research Center, Radiation Oncology Ward, Cancer Institute, IKHC, Qarib Street, Tehran, Iran

**Keywords:** Rectal cancer, Oxaliplatin, Radiotherapy, Survival, Neoadjuvant therapy

## Abstract

**Background/Aim:**

Current approaches for locally advanced rectal cancer (LARC) typically recommend neoadjuvant chemoradiotherapy (nCRT) with 5-fluorouracil (5FU) or its oral analogs followed by surgery as the standard of care. However, the question of whether intensifying concurrent chemotherapy by adding oxaliplatin to the 5FU-based backbone can yield better outcomes remains unresolved. This study aimed to investigate the benefits of incorporating oxaliplatin into fluoropyrimidine-based chemoradiotherapy (CRT) to increase locoregional control and survival.

**Methods:**

Among 290 patients with LARC admitted to the Iran Cancer Institute’s radiation oncology department between January 2008 and December 2019, 29 received CAPEOX (capecitabine 625 mg/m²/bid on RT days and weekly oxaliplatin 50 mg/m²), whereas 293 received capecitabine (825 mg/m² twice daily or rarely 5FU in the first 4 days and last week of radiotherapy (RT)). Variables potentially affecting treatment outcomes were used for propensity score matching. Kaplan‒Meier and log-rank tests were employed for overall survival (OS) and disease-free survival (DFS) analyses and were adjusted with propensity score matching.

**Results:**

Data from 29 patients who received CAPEOX and 216 patients who received capecitabine were analyzed after propensity score matching without replacement. After propensity score matching, in the multivariate analysis, CAPEOX significantly increased the likelihood of achieving a pathologic complete response (pCR) by 4.38 times (CI: 1.90–10.08, p value < 0.001). However, CAPEOX did not demonstrate any statistically significant predictive value for DFS (*P* = 0.500) or OS (*P* = 0.449).

**Conclusion:**

The addition of oxaliplatin resulted in a significantly higher rate of pCR without any translation into long-term survival outcomes.

## Background

According to the latest GLOBOCAN data, over 1.9 million new cases of colorectal cancer and over 930,000 deaths were estimated to have occurred worldwide in 2022. Overall, colorectal cancer (CRC) is ranked third in terms of incidence but second in terms of mortality. Rectal cancer comprises approximately 30% of all colorectal cancers [1]. The optimal management of LARC has changed dramatically in recent years. Historically, treatment typically involved neoadjuvant long-course chemoradiotherapy or short-course radiotherapy followed by surgery and postoperative chemotherapy at the discretion of the physician [2]. However, owing to the promising results of total neoadjuvant therapy (TNT), as recommended by the National Comprehensive Cancer Network (NCCN), the standard treatment for LARC includes a TNT approach involving both chemoradiation and chemotherapy courses given as neoadjuvant therapy and then surgery or a watch and wait for complete clinical responders who wish to pursue nonoperative management [3].

Although the combination of fluorouracil or capecitabine with oxaliplatin forms the cornerstone of chemotherapy regimens for induction or consolidation purposes, both the NCCN and European Society for Medical Oncology (ESMO) guidelines recommend the use of 5FU or capecitabine alone with long-course radiotherapy as neoadjuvant therapy for rectal cancer [3, 4]. One of the main reasons for deviations from the ESMO clinical guidelines in some real-world settings is the addition of oxaliplatin. Some scientists believe that in patients with high-risk factors, such as the presence of positive extramesorectal lymph nodes, tumor deposits, and extramural vascular invasion (EMVI), the intensification of concomitant chemotherapy with oxaliplatin in addition to fluoropyrimidine can be considered [5, 6]. While achieving a pCR or locoregional control (LRC) and survival are the main endpoints for many clinical trials, several studies have investigated the advantages of adding oxaliplatin to fluoropyrimidine-based CRT to improve short-term or long-term oncological outcomes. The results were mixed, while the majority of phase III trials did not demonstrate a clear advantage for adding oxaliplatin [7–9], some phase III and numerous phase II trials reported advantages in terms of pCR, DFS, and LRC [10–14]. Additionally, meta-analyses have yielded mixed results, suggesting improvements in pCR and outcomes related to distant metastasis but no impact on LRC or survival [15, 16].

An increase in pCR at the cost of increased toxicity, mainly neuropathy, and no effect on LRC or OS are common findings in the majority of studies [17]. With the advent of new endpoints such as total mesorectal excision (TME)-free survival as a goal for nonoperative management, achieving a sustained response is more important than before [18]. Thus, the idea of studying agents such as oxaliplatin that might have a beneficial effect on increasing the response to neoadjuvant therapy merits a revisit.

Given these inconsistent findings, this study aimed to assess and elucidate the actual benefit of adding oxaliplatin to neoadjuvant chemoradiation for LARC outcomes in a real-world setting.

## Methods

### Study population

In this retrospective cohort study, we obtained records of patients diagnosed with stage II and III rectal cancer with the epicenter of the tumor below the sacral promontory, referred to the radiation oncology department of the Iran Cancer Institute in Tehran, Iran, between January 2008 and December 2019.

Data, including sex, age, treatment type, clinical stage, RT interval to surgery, lowest distance to anal verge (AV), adjuvant therapy, and patient status at the last follow-up, were retrieved.

### Treatment specifications

The preoperative staging work-up comprised a comprehensive total colonoscopy with biopsies from all suspicious sites; routine laboratory tests, including carcinoembryonic antigen (CEA) level assessment; gadolinium-enhanced pelvic magnetic resonance imaging (MRI); and/or endoscopic ultrasonography. Additionally, all patients underwent contrast-enhanced thoracoabdominal and pelvic computed tomography (CT) scans.

All patients underwent neoadjuvant chemoradiation RT involved delivering either 50 Gy in 25 daily fractions in one phase or 45 Gy to the whole pelvis, followed by an additional 5.4 Gy boost to the mesorectum in daily fractions of 1.8 Gy over 5.5 weeks. The determination of the appropriate radiotherapy dosage was at the discretion of the attending physician. All patients were planned using 3D conformal technique. the target delineation was based on the International consensus study by Valentini et al. published in 2016 [19]. During radiotherapy, patients received either oral capecitabine at a dose of 825 mg/m^2^ twice daily alone or CAPEOX, including oxaliplatin 50 mg/m^2^/weekly and capecitabine 625 mg/m^2^ twice daily. Capecitabine was administered only on radiotherapy days in both groups. Oxaliplatin was given for 4‒5 cycles. The administration of each regimen was at the discretion of the attending physician without following special criteria.

All patients were initially planned to undergo TME, with the most commonly employed techniques being low anterior resection (LAR) and abdominoperineal resection (APR) for low-lying tumors that are not suitable for organ-sparing procedures. All patients underwent Open surgery. The quality of TME was not assessable. The selection of the suitable technique was left to the judgment of the attending surgeon overseeing the surgery.

### Postoperative outcomes and follow-up

Pathological staging was revised in accordance with the American Joint Committee on Cancer (AJCC) 8^th^ edition staging system during data review [20]. The extent of tumor regression in pathology reports was uniformly recorded via the 2010 AJCC tumor regression grading (TRG) system [21].

Patients with pathologic nodal metastases, positive resection margins, or pathologic T3-T4 tumors received adjuvant chemotherapy. Other patients were administered adjuvant chemotherapy with mFOLFOX or CAPEOX at the discretion of their attending physicians. We did not have access to the adjuvant chemotherapy protocols. Institutional follow-up included physical examination and a serum CEA assay every three months for the first two years, followed by assessments every six months (CEA testing was discontinued after the 5th year); chest/abdominopelvic CT scans every six months for the initial three years, then annually until the fifth year; and colonoscopy at 1-, 3-, and 5-years post-surgery if the results were normal. Additional tests, such as pelvic MRI or positron emission tomography scans, were requested as indicated. This study adheres to the STROBE statement for cohort studies [22].

The primary outcome assessed was the achievement of pCR. pCR was defined as no residual disease in the bed of the primary tumor and dissected lymph nodes (ypT0N0). The secondary outcomes included OS and DFS. OS was measured from the termination of radiotherapy to the date of death or last follow-up; DFS was measured from the termination of radiotherapy to the occurrence of locoregional or distant recurrence or death attributable to any cause or until the last uneventful follow-up for survivors. Local recurrence was detected and confirmed by DRE and endoscopic examination. Distant metastasis was diagnosed and confirmed through radiological identification of enlarging lesions (using abdominopelvic and thorax CT-scan and in some cases pelvic MRI and PET-CT) with or without histologic confirmation.

### Statistical analysis

Descriptive data analysis was employed to determine frequencies (percentages) or means and standard deviations for categorical and quantitative data, respectively. To compare the rates of pCR between the groups, the chi-square test and multivariate logistic regression were utilized. Kaplan‒Meier survival analysis was conducted to estimate the OS and DFS rates. The log-rank test was used to compare survival between groups. Cox regression was used to calculate hazard ratios (HRs) between survival curves.

Variables potentially influencing pCR were used to generate propensity score matching. The associations between the concurrent chemotherapy regimens and survival endpoints, including pCR, OS, and DFS, were evaluated before and after adjustment with propensity score matching. Propensity score matching was performed with a 0.01 caliper and without replacement, utilizing the following variables: RT interval to surgery, lowest distance to the AV, clinical stage, age, sex and adjuvant therapy. The collected data were input and analyzed via STATA software (Version 23, IBM, Chicago, IL). A significance threshold was set at p values below 0.05.

It is important to note that the restricted sample size, particularly within the CAOEOX group, elevates the likelihood of a type II error, which may subsequently impact the statistical analysis’s power.

### Ethics statement

This study design was approved by the institutional review board (code: 1401-4-417-63804) and the ethics committee of Tehran University of Medical Sciences (IR.TUMS.IKHC.REC.1401.397). The present study was conducted in accordance with the principles outlined in the Declaration of Helsinki. Before their inclusion in the study, all patients had provided written informed consent regarding the use of their personal data for research purposes upon admission.

## Results

The patients’ characteristics are reported in Table 1. Before propensity score matching, out of 290 patients, 29 received CAPEOX, and 261 received capecitabine. After propensity score matching, 216 patients remained in the capecitabine group. Men accounted for 72.41% of the patients who received CAPEOX, whereas in the capecitabine group, 59% and 62.5% of the patients were male before and after propensity score matching, respectively. The mean age of patients who received CAPEOX was 49.8 years (SD: 14.3), whereas the mean age of those who received capecitabine was greater. Details of the other characteristics, including the lowest distance to the AV, clinical stage, and RT interval to surgery, are shown in Table 1.

**Table 1:** Patients’ characteristics

Variables, *n* (%)	Before Propensity Score Matching		After Propensity Score Matching	
	CAPEOX (*n* = 29)	Capecitabine (*n* = 261)	CAPEOX (*n* = 29)	Capecitabine (*n* = 216)
**Sex**				
Male	21 (72.41)	154 (59.00)	21 (72.41)	135 (62.50)
Female	8 (27.59)	107 (41.00)	8 (27.59)	81 (37.50)
**Age**, **mean (SD)**	49.8 (14.3)	56.6 (12.2)	49.8 (14.3)	55.7 (12.1)
**Lowest distance to AV** (cm)				
≤ 5	18 (62.07)	113 (43.30)	18 (62.07)	102 (47.22)
5 < ≤ 10	9 (31.03)	109 (41.76)	9 (31.03)	95 (43.98)
> 10	2 (6.90)	34 (13.03)	2 (6.90)	19 (8.80)
Unknown	0 (0)	5 (1.92)	0 (0)	0 (0)
**Clinical stage**				
II	5 (17.24)	24 (9.20)	5 (17.24)	22 (10.19)
III	20 (68.97)	181 (69.35)	20 (68.97)	152 (70.37)
Unknown	4 (13.79)	56 (21.46)	4 (13.79)	42 (19.44)
**RT Interval to Surgery** (weeks)				
< 12	20 (68.97)	144 (55.17)	20 (68.97)	127 (58.80)
≥ 12	9 (31.03)	117 (44.83)	9 (31.03)	89 (41.20)
**Adjuvant therapy**				
No	9 (31.03)	84 (32.18)	9 (31.03)	67 (31.02)
Yes	20 (68.97)	170 (65.13)	20 (68.97)	144 (66.67)
Unknown	0 (0)	7 (2.68)	0 (0)	5 (2.31)

Data are presented as number (%) and mean (± SD). Abbreviations: AV, anal verge; RT, radiotherapy

The results of logistic regression analysis investigating factors associated with the primary outcome, pCR, are presented in Table 2. Among patients who received capecitabine, 21.76% achieved pCR, whereas 55.17% achieved pCR among those who received CAPEOX. Before propensity score matching, among all the factors, only CAPEOX was significantly associated with the odds of achieving pCR (OR: 4.68, CI: 2.03–10.78, p value < 0.001). This association remained consistent after propensity score matching, with CAPEOX being the sole factor significantly increasing the likelihood of achieving pCR by 4.38 times in the multivariate analysis (CI: 1.90–10.08, p value < 0.001).

**Table 2:** Logistic regression of factors associated with odds of pCR

	Before Propensity Score Matching				After Propensity Score Matching			
Variables (Reference Level)	Univariate analysis		Multivariate analysis		Univariate analysis		Multivariate analysis	
	OR (95% CI)	P- Value	OR (95% CI)	P- Value	OR (95% CI)	P-Value	OR (95% CI)	P-Value
**Sex** (male)	1.06 (0.61–1.84)	0.814	1.20 (0.67–2.13)	0.534	1.01 (0.55–1.83)	0.972	1.12 (0.59–2.10)	0.722
**Age**	0.99 (0.97–1.01)	0.684	1.00 (0.98–1.02)	0.762	0.99 (0.97–1.01)	0.627	1.00 (0.97–1.02)	0.954
**Lowest distance to AV** (≤ 5 cm)								
5 < distance ≤ 10 cm	0.84 (0.47–1.51)	0.574	0.99 (0.54–1.84)	0.997	0.87 (0.47–1.59)	0.653	1.02 (0.53–1.93)	0.949
> 10 cm	1.09 (0.47–2.50)	0.826	1.55 (0.64–3.75)	0.323	1.10 (0.39–3.07)	0.856	1.45 (0.48–4.35)	0.502
**Clinical stage** (II) III	0.81 (0.35–1.90)	0.638	0.98 (0.40–2.42)	0.974	0.75 (0.31–1.79)	0.520	0.86 (0.34–2.17)	0.759
**RT Interval to Surgery** (< 12 weeks)	0.86 (0.50–1.49)	0.611	0.90 (0.51–1.59)	0.727	0.82 (0.45–1.48)	0.512	0.88 (0.47–1.63)	0.695
**Adjuvant therapy** (No)	1.22 (0.68–2.19)	0.49	1.32 (0.71–2.44)	0.360	1.22 (0.65–2.29)	0.539	1.30 (0.67–2.52)	0.432
**CAPEOX** (Capecitabine)	4.60 (2.09–10.15)	< 0.001	4.68 (2.03–10.78)	< 0.001	4.42 (1.98–9.84)	< 0.001	4.38 (1.90-10.08)	0.001

Bold numbers indicate statistical significance (*p* < 0.05). Abbreviations: AV, anal verge; RT, radiotherapy; CI, confidence interval; OR, odds ratio; pCR, pathologic complete response

The median follow-up time was 44 and 47 months before and after propensity score matching, respectively. The median OS and DFS were 41 and 34 months, respectively, for patients who received capecitabine. For patients who received CAPEOX, the median OS and DFS were 57 and 33 months, respectively. The 3- and 5-year OS rates were 78% and 76% and 51% and 67%, respectively, in the capecitabine and CAPEOX groups. The 3- and 5-year DFS rates were 64% vs. 76% and 40% vs. 67% in the capecitabine and CAPEOX groups, respectively. The results of Cox proportional hazards regression, aimed at estimating hazard ratios (HRs) for prognostic risk factors affecting DFS, are summarized in Table 3. Before propensity score matching, among all the factors, only pCR was significantly associated with DFS (HR: 0.25, CI: 0.13–0.48, p value < 0.001). This association remained consistent after propensity score matching (HR: 0.26, CI: 0.13–0.51, p value < 0.001). Adding oxaliplatin did not have any statistically significant predictive value for DFS.

**Table 3:** HR estimation for prognostic risk factors on DFS

	Before Propensity Score Matching				After Propensity Score Matching			
Variables (Reference Level)	Univariate analysis		Multivariate analysis		Univariate analysis		Multivariate analysis	
	HR (95% CI)	P- Value	HR (95% CI)	P Value	HR (95% CI)	P Value	HR (95% CI)	P Value
**Sex** (male)	1.06 (0.73–1.56)	0.728	1.00 (0.68–1.47)	0.989	1.06 (0.69–1.62)	0.775	0.95 (0.61–1.46)	0.821
**Age**	1.01 (0.99–1.03)	0.066	1.01 (0.99–1.03)	0.070	1.01 (0.99–1.02)	0.326	1.00 (0.99–1.02)	0.332
**Lowest distance to AV** (≤ 5 cm)								
5 < ≤ 10	1.14 (0.76–1.70)	0.507	1.11 (0.74–1.67)	0.601	1.22 (0.80–1.86)	0.353	1.15 (0.74–1.77)	0.517
> 10	0.72 (0.36–1.43)	0.357	0.67 (0.33–1.36)	0.276	0.74 (0.31–1.74)	0.497	0.62 (0.25–1.49)	0.289
**Clinical stage** (II) III	0.72 (0.40–1.31)	0.290	0.57 (0.31–1.06)	0.077	0.69 (0.37–1.29)	0.256	0.56 (0.29–1.06)	0.075
**RT Interval to Surgery** (< 12 weeks)	1.12 (0.77–1.64)	0.533	1.13 (0.76–1.67)	0.523	1.06 (0.69–1.61)	0.784	1.00 (0.65–1.54)	0.966
**Adjuvant therapy** (No)	0.81 (0.54–1.20)	0.294	0.82 (0.55–1.22)	0.320	0.82 (0.53–1.28)	0.393	0.85 (0.54–1.33)	0.481
**CAPEOX** (Capecitabine)	0.74 (0.39–1.39)	0.354	1.29 (0.66–2.50)	0.445	0.76 (0.40–1.43)	0.401	1.25 (0.64–2.46)	0.500
**pCR**	0.26 (0.14–0.47)	**< 0.001**	0.25 (0.13–0.48)	**< 0.001**	0.27 (0.14–0.52)	**< 0.001**	0.26 (0.13–0.51)	**< 0.001**

Bold numbers indicate statistical significance (*p* < 0.05). Abbreviations: AV, anal verge; RT, radiotherapy; CI, confidence interval; HR, hazard ratio; pCR, pathologic complete response; DFS, disease-free survival

The results of Cox proportional hazards regression for prognostic risk factors affecting OS are summarized in Table 4. Before propensity score matching, among all the factors, only pCR was significantly associated with OS (HR: 0.25, CI: 0.12–0.51, p value < 0.001). This association remained consistent after propensity score matching (HR: 0.25, CI: 0.12–0.54, p value < 0.001).

**Table 4:** HR estimation for prognostic risk factors on OS

	Before Propensity Score Matching				After Propensity Score Matching			
Variables (Reference Level)	Univariate analysis		Multivariate analysis		Univariate analysis		Multivariate analysis	
	HR (95% CI)	P- Value	HR (95% CI)	P-Value	HR (95% CI)	P- Value	HR (95% CI)	P- Value
**Sex** (male)	1.09 (0.71–1.66)	0.669	1.04 (0.68–1.60)	0.831	1.09 (0.68–1.75)	0.702	0.98 (0.61–1.59)	0.965
**Age**	1.02 (0.99–1.03)	0.065	1.01 (0.99–1.03)	0.071	1.01 (0.99–1.03)	0.281	1.00 (0.99–1.02)	0.299
**Lowest distance to AV** (≤ 5 cm)								
5 < ≤ 10	1.18 (0.76–1.83)	0.452	1.13 (0.72–1.77)	0.582	1.17 (0.73–1.86)	0.504	1.09 (0.68–1.76)	0.696
> 10	0.64 (0.29–1.44)	0.288	0.61 (0.27–1.40)	0.252	0.61 (0.22–1.73)	0.360	0.55 (0.19–1.61)	0.283
**Clinical stage** (II) III	0.82 (0.42–1.62)	0.583	0.64 (0.32–1.28)	0.212	0.75 (0.38–1.49)	0.422	0.61 (0.30–1.24)	0.179
**RT Interval to Surgery** (< 12 weeks)	1.12 (0.73–1.71)	0.588	1.06 (0.68–1.63)	0.788	1.10 (0.69–1.75)	0.686	1.01 (0.63–1.62)	0.958
**Adjuvant therapy** (No)	0.82 (0.52–1.28)	0.378	0.83 (0.53–1.32)	0.434	0.76 (0.47–1.24)	0.287	0.81 (0.49–1.34)	0.424
**CAPEOX** (Capecitabine)	0.74 (0.37–1.50)	0.415	1.34 (0.64–2.80)	0.434	0.77 (0.38–1.56)	0.475	1.33 (0.63–2.82)	0.449
**pCR**	0.26 (0.13–0.51)	**< 0.001**	0.25 (0.12–0.51)	**< 0.001**	0.27 (0.13–0.56)	**< 0.001**	0.25 (0.12–0.54)	**< 0.001**

Bold numbers indicate statistical significance (*p* < 0.05). Abbreviations: AV, anal verge; RT, radiotherapy; CI, confidence interval; HR, hazard ratio; pCR, pathologic complete response; OS, overall survival

**Fig. 1:**
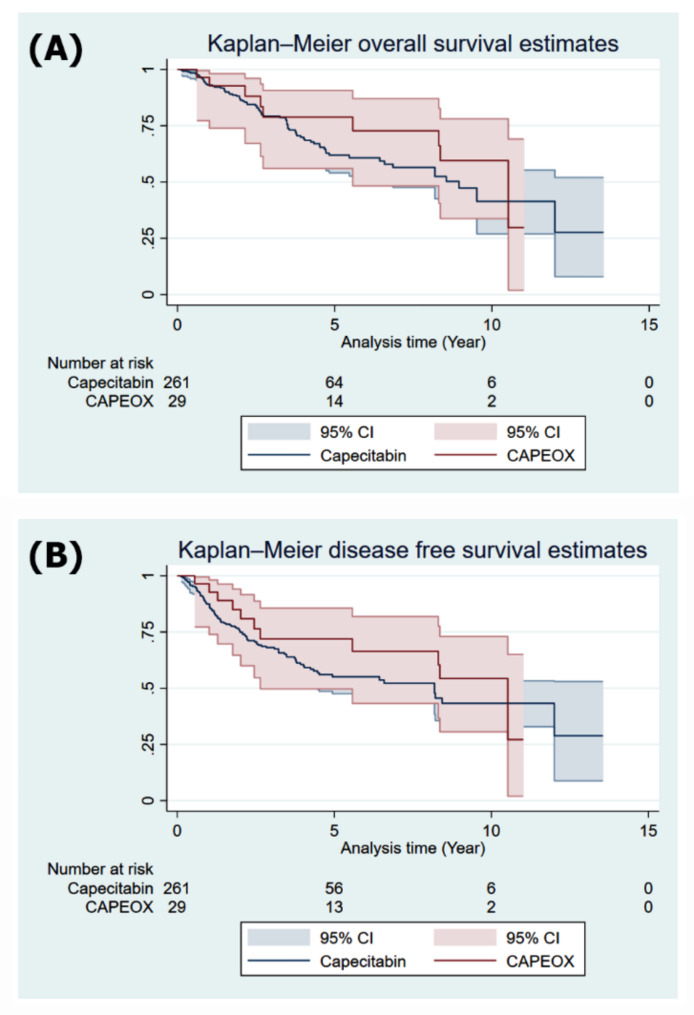
Kaplan‒Meier (K-M) survival analysis before propensity score matching. (**A**) K‒M survival analysis for overall survival (OS). (**B**) K‒M survival analysis for disease free survival (DFS)

Adding oxaliplatin did not have any statistically significant predictive value for OS. The Fig. 1 shows the results of the Kaplan‒Meier survival analysis and log-rank analyses for OS and DFS before propensity score matching. Patients who received CAPEOX and those who received capecitabine had similar OS and DFS rates.

**Fig. 2:**
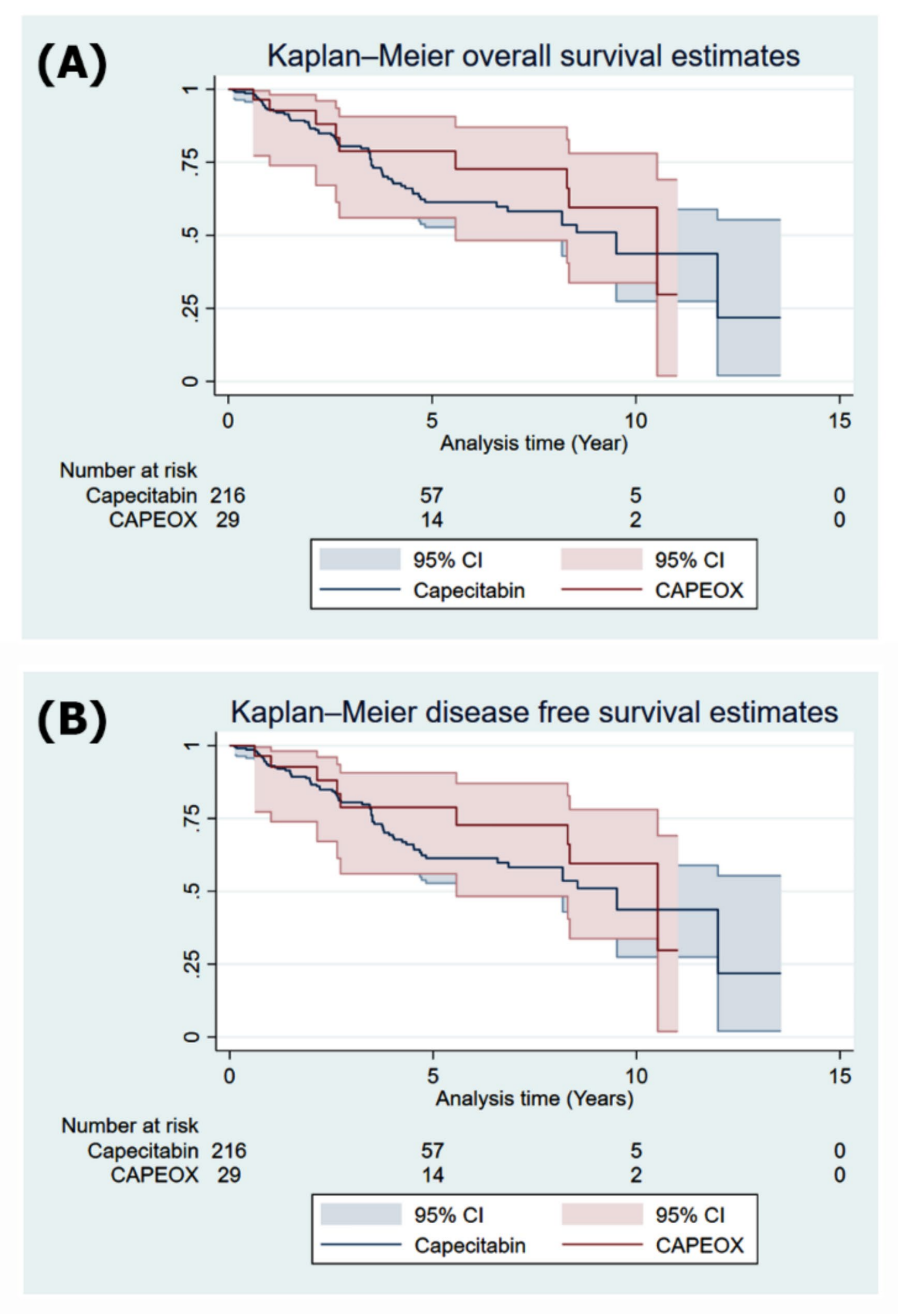
Kaplan‒Meier (K-M) survival analysis after propensity score matching. (**A**) K‒M survival analysis for overall survival (OS). (**B**) K‒M survival analysis for disease free survival (DFS)

Furthermore, the same analysis was performed after propensity score matching, as shown in Fig. 2. The OS and DFS rates were not significantly different between the two treatment groups.

## Discussion

In this retrospective study, we assessed the potential advantages of adding oxaliplatin to routine nCRT for patients with LARC via a propensity score-matched design. The findings revealed that patients who received oxaliplatin achieved significantly higher rates of pCR as a short-term outcome, with no statistically significant differences in longer-term outcomes, including OS and DFS.

There are discrepancies among studies that reported the outcomes after using oxaliplatin as a component of concurrent chemoradiotherapy in rectal cancer. To achieve a comprehensive understanding of the findings presented in various literature, the outcomes of the articles are classified into two distinct categories: those that yield favorable results and those that produce unfavorable results. In the NSABP R-04 trial [8], the inclusion of oxaliplatin did not lead to improved oncological outcomes. The PETACC6 trial [7] similarly found no advantages in 5-year disease-free survival (DFS), overall survival (OS), or local recurrence control (LRC) when comparing CAPEOX to capecitabine followed by surgery and adjuvant CAPEOX. The STAR-01 trial [9] also reported no significant tumor response with the addition of oxaliplatin to preoperative neoadjuvant chemoradiotherapy (nCRT), and unexpectedly observed an increase in early distant recurrence [23]. The ACCORD12 trial [24] produced comparable results, showing no differences in DFS or OS between capecitabine alone and the combination of capecitabine with oxaliplatin. Additionally, the INTERACT trial [11], which utilized intensified chemotherapy, did not demonstrate any differences in pathological complete response (pCR), 5-year OS, or DFS. At least two other studies also showed negative results and failed to demonstrate any benefit with the addition of oxaliplatin to capecitabine or fluorouracil [25, 26].

In contrast some studies showed promising findings when oxaliplatin added to the conventional capecitabine/5FU-based chemoradiotherapy. In a comparative analysis of different treatment protocols in phase III trials, the FOWARC study [13] demonstrated that the incorporation of oxaliplatin into mFOLFOX CRT yielded a superior pathologic complete response (pCR) rate relative to 5FU-based therapies; however, this enhancement did not translate into improved overall survival rates. Conversely, the CAO/ARO/AIO-04 trial [14] revealed that the addition of oxaliplatin to concurrent 5FU resulted in an increase in disease-free survival (DFS) without affecting overall survival (OS), particularly among patients who achieved pCR. Haddad et al. also reported an elevated pCR rate associated with oxaliplatin in the context of neoadjuvant therapy [27] which is consistent with our finding. In another recent report, researchers discovered that TNT (RT with two concurrent cycles of capecitabine and oxaliplatin (CAPEOX) followed by another two cycles of CAPEOX) resulted in a higher rate of pCR and 3-year DFS than did nCRT (RT with concurrent capecitabine) [28]. Meta-analyses conducted by various scholars have concluded that while oxaliplatin is associated with increased pCR rates and a reduction in distant recurrence, it does not significantly influence overall survival, disease-free survival, or locoregional control (LRC). In the study by Hüttner et al. [15], which included 5,599 patients, no advantage was observed for adding oxaliplatin in terms of OS, DFS, or LRC. However, it resulted in an increase in pCR rates (OR = 1.31, *P* = 0.002) and a reduction in distant recurrence. Fu et al. [16], in their analysis involving 6103 patients, reported that patients who received oxaliplatin achieved higher rates of pCR (OR = 1.29, *P* = 0.0005) and 3-year DFS, with no differences in OS. According to another meta-analysis by De Felice et al. in 2017 involving 4 RCTs [12], patients treated with oxaliplatin experienced decreased rates of distant failure, although OS, DFS, and LRC did not differ. The *Hoerndervangers* indicated that the inclusion of oxaliplatin may lead to a higher rate of pCR, although this advantage does not translate into improved survival [29], which is consistent with the results of our study. The prevailing consensus indicates that the addition of oxaliplatin may enhance short-term outcomes such as pCR, yet it does not improve overall survival.

When discussing the studies in this regard we should also note that the efficacy of oxaliplatin is influenced by factors such as dosage. Most trials adopted a weekly dosage of 50 mg/m2 alongside 5FU-based radiotherapy. Nonetheless, some studies have employed alternative schedules. In the FOWARC trial, patients underwent five 2-week cycles of infusional 5FU and oxaliplatin, followed by surgery. The oxaliplatin dosage was 130 mg/m2 administered at weeks 1 and 5 of RT, resulting in a moderate response rate [30]. Greto et al. investigated a protocol in 2013 involving nCRT with oxaliplatin and 5FU, followed by surgery. The oxaliplatin dosage was 80 mg/m2 at weeks 1 and 5, resulting in favorable outcomes for both OS and pCR [31]. Lee et al. administered nCRT with oxaliplatin and 5FU, followed by surgery, with an oxaliplatin dosage of 130 mg/m2 administered at weeks 1 and 5. However, unlike the aforementioned trials, no improvements in pCR were observed [32]. Chang et al. reported that patients receiving a cumulative oxaliplatin dose less than 460 mg/m2 experienced poorer OS and DFS [33]. Jiao et al. and the ADORE trial administered cumulative doses of oxaliplatin at 680 mg/m2 and 750–920 mg/m2, respectively, demonstrating the superiority of incorporating oxaliplatin regimens in improving 3-year DFS [34, 35]. Trials reporting negative outcomes typically administer lower cumulative oxaliplatin doses (STAR-01 360 mg/m2, ACCORD12 250 mg/m2, and NSABP R-04 250 mg/m2), followed by surgery and subsequent seven cycles of mFOLFOX chemotherapy. This protocol, with an oxaliplatin dosage of 85 mg/m2 administered concurrently with RT for three cycles, yielded improvements in pCR [13]. Tang et al. treated 45 patients in 2018 with nCRT comprising oxaliplatin and capecitabine, followed by surgery and CAPEOX cycles. Unlike the prevailing weekly schedule, oxaliplatin was administered at 130 mg/m2 on weeks 1 and 3 [36].

One important point of view that may justify the renewed interest in the addition of oxaliplatin to conventional capecitabine/5FU-based chemoradiotherapy as an intensified neoadjuvant regimen is non-operative management (NOM) and organ preservation. In this context, patients who show a complete clinical response via proctoscopy and MRI can postpone or forego surgery until the tumor regrows. The OPRA trial was one of the main studies in this regard, showing that a considerable proportion of patients are candidates for such treatment [37]. Thus, the use of concurrent oxaliplatin as a means of increasing the response to chemoradiotherapy is a viable option.

The available literature suggests that by adding oxaliplatin there are some short-term benefits in enhancing response to nCRT, however, without any translated improvements in long-term outcomes including overall and DFS. Thus, the challenge of oxaliplatin use has not been resolved completely in the literature. It needs further investigation, especially in some subgroups with rectal cancer. These may include patients with excellent to good performance status with low-lying tumors requiring sphincter preservation or synchronous resectable metastases. Oxaliplatin can also be considered for patients with excellent performance status who have tumors with a high risk of failure [17].

This study has certain limitations. First, given its retrospective nature, the findings may only become apparent after an extended follow-up period. Second, variations among patients receiving adjuvant CAPEOX or capecitabine therapy could have influenced outcomes; however, we did not have access to the adjuvant chemotherapy medications administered by patients, but we adjusted the receipt of adjuvant chemotherapy overall. Third, this study does not address the toxicity associated with concurrent oxaliplatin, as this toxicity might reduce the tolerability of patients for adjuvant therapy and the optimal timing of surgery in poor responders to radiotherapy, thereby limiting the ability to assess the risk-benefit ratio of incorporating oxaliplatin.

## Conclusion

The results of the present study suggest that adding oxaliplatin to nCRT is beneficial for achieving pCR. However, these enhancements do not translate into improved long-term survival outcomes, including OS and DFS. Nonetheless, oxaliplatin may still be considered for patients who prefer alternative treatment approaches, such as organ preservation or nonoperative management. Our findings corroborate the potential of oxaliplatin in increasing the likelihood of achieving pCR. Particularly in cases where tumor size necessitates preoperative reduction, oxaliplatin can serve as a viable adjunct, albeit without conferring substantial long-term survival benefits. In the future, multicentric investigations on a larger population are needed to fully resolve the challenges associated with the use of oxaliplatin and to better describe the early outcomes and survival patterns across all subtypes of patients with rectal cancer. In the nonoperative management era, the addition of oxaliplatin to conventional concurrent chemoradiotherapy merits a revisit.

## Data Availability

The data that support the findings of this study are available from the corresponding author upon reasonable request.

## Abbreviations

LARC: Locally advanced rectal cancer
nCRT: Neoadjuvant chemoradiotherapy
5FU: 5-fluorouracil
CRT: Chemoradiotherapy
RT: Radiotherapy
OS: Overall survival
DFS: Disease-free survival
pCR: Pathologic complete response
CRC: Colorectal cancer
TNT: Total neoadjuvant therapy
NCCN: NATIONAL Comprehensive Cancer Network
ESMO: EUROPEAN Society for Medical Oncology
EMVI: Extramural vascular invasion
LRC: Locoregional control
TME: Total mesorectal excision
AV: Anal verge
CEA: Carcinoembryonic antigen
CT: Computed tomography
LAR: Low anterior resection
APR: Abdominoperineal resection
AJCC: American Joint Committee on Cancer
TRG: Tumor regression grading
HRs: Hazard ratios
NOM: Nonoperative management

## References

[1] Bray F, Laversanne M, Sung H, Ferlay J, Siegel RL, Soerjomataram I, Jemal A. Global cancer statistics 2022: GLOBOCAN estimates of incidence and mortality worldwide for 36 cancers in 185 countries. CA: a cancer journal for clinicians. 2021. [13 May 2024]; https://www.nccn.org/professionals/physician_gls/pdf/rectal.pdf 10.3322/caac.21834 38572751

[2] Aghili M, et al. Short-course versus long-course neoadjuvant chemoradiotherapy in patients with rectal cancer: preliminary results of a randomized controlled trial. Radiat Oncol J. 2020;38(2):119–28. 33012155 10.3857/roj.2020.00115 PMC7533412

[3] NCCN Clinical Practice Guidelines on Oncology: Rectal Cancer. Version 2.2024. 2024 May 14. 2024]; https://www.nccn.org/guidelines/guidelines-detail?category=1&id=1461

[4] Glynne-Jones R, et al. Rectal cancer: ESMO Clinical Practice guidelines for diagnosis, treatment and follow-up. Ann Oncol. 2017;28:iv22–40. 28881920 10.1093/annonc/mdx224

[5] Savino M, et al. A process mining approach for clinical guidelines compliance: real-world application in rectal cancer. Front Oncol. 2023;13:1090076. 37265796 10.3389/fonc.2023.1090076 PMC10231435

[6] Bahadoer RR, et al. Short-course radiotherapy followed by chemotherapy before total mesorectal excision (TME) versus preoperative chemoradiotherapy, TME, and optional adjuvant chemotherapy in locally advanced rectal cancer (RAPIDO): a randomised, open-label, phase 3 trial. Lancet Oncol. 2021;22(1):29–42. 33301740 10.1016/S1470-2045(20)30555-6

[7] Schmoll H-J, et al. Pre-and postoperative capecitabine without or with oxaliplatin in locally advanced rectal cancer: PETACC 6 trial by EORTC GITCG and ROG, AIO, AGITG, BGDO, and FFCD. J Clin Oncol. 2021;39(1):17–. 33001764 10.1200/JCO.20.01740

[8] Allegra CJ, et al. Neoadjuvant 5-FU or capecitabine plus radiation with or without oxaliplatin in rectal cancer patients: a phase III randomized clinical trial. J Natl Cancer Inst. 2015;107(11):djv248. 26374429 10.1093/jnci/djv248 PMC4849360

[9] Aschele C et al. Final results of STAR-01: A randomized phase III trial comparing preoperative chemoradiation with or without oxaliplatin in locally advanced rectal cancer. 2016, American Society of Clinical Oncology. 10.1200/JCO.2010.34.4911 21606427

[10] Yu X, et al. Neoadjuvant oxaliplatin and capecitabine combined with bevacizumab plus radiotherapy for locally advanced rectal cancer: results of a single-institute phase II study. Cancer Commun. 2018;38:1–9. 10.1186/s40880-018-0294-z PMC5993137 29784042

[11] Valentini V, et al. The INTERACT Trial: long-term results of a randomised trial on preoperative capecitabine-based radiochemotherapy intensified by concomitant boost or oxaliplatin, for cT2 (distal)–cT3 rectal cancer. Radiother Oncol. 2019;134:110–8. 31005204 10.1016/j.radonc.2018.11.023

[12] De Felice F, et al. Clinical benefit of adding oxaliplatin to standard neoadjuvant chemoradiotherapy in locally advanced rectal cancer: a meta-analysis: oxaliplatin in neoadjuvant treatment for rectal cancer. BMC Cancer. 2017;17:1–6. 28499428 10.1186/s12885-017-3323-4 PMC5427623

[13] Deng Y, et al. Modified FOLFOX6 with or without radiation versus fluorouracil and leucovorin with radiation in neoadjuvant treatment of locally advanced rectal cancer: initial results of the Chinese FOWARC multicenter, open-label, randomized three-arm phase III trial. J Clin Oncol. 2016;34(27):3300–7. 27480145 10.1200/JCO.2016.66.6198

[14] Rödel C, et al. Oxaliplatin added to fluorouracil-based preoperative chemoradiotherapy and postoperative chemotherapy of locally advanced rectal cancer (the German CAO/ARO/AIO-04 study): final results of the multicentre, open-label, randomised, phase 3 trial. Lancet Oncol. 2015;16(8):979–89. 26189067 10.1016/S1470-2045(15)00159-X

[15] Huettner FJ, et al. Addition of platinum derivatives to neoadjuvant single-agent fluoropyrimidine chemoradiotherapy in patients with stage II/III rectal cancer: protocol for a systematic review and meta-analysis (PROSPERO CRD42017073064). Syst Reviews. 2018;7:1–7. 10.1186/s13643-018-0678-9 PMC5778669 29357929

[16] Fu X-L, et al. Meta-analysis of oxaliplatin-based versus fluorouracil-based neoadjuvant chemoradiotherapy and adjuvant chemotherapy for locally advanced rectal cancer. Oncotarget. 2017;8(21):34340. 28423720 10.18632/oncotarget.16127 PMC5470972

[17] Nazari R, et al. Role of Oxaliplatin in the Neoadjuvant Concurrent Chemoradiotherapy in locally advanced rectal Cancer: a review of evidence. Clin Med Insights: Oncol. 2024;18:11795549241236409. 38510317 10.1177/11795549241236409 PMC10952988

[18] Romero Zoghbi S et al. Total neoadjuvant therapy approach for the treatment of locally advanced rectal cancer. Where do we stand? 2023. 10.1159/000534888 PMC11216349 37935161

[19] Valentini V, et al. International consensus guidelines on clinical target volume delineation in rectal cancer. Radiother Oncol. 2016;120(2):195–201. 27528121 10.1016/j.radonc.2016.07.017

[20] Amin MB, et al. The eighth edition AJCC cancer staging manual: continuing to build a bridge from a population-based to a more personalized approach to cancer staging. Cancer J Clin. 2017;67(2):93–9. 10.3322/caac.21388 28094848

[21] Edge SB, Compton CC. The American Joint Committee on Cancer: the 7th edition of the AJCC cancer staging manual and the future of TNM. Annals of surgical oncology, 2010. 17(6): pp. 1471–1474. 10.1245/s10434-010-0985-4 20180029

[22] Von Elm E, et al. The strengthening the reporting of Observational studies in Epidemiology (STROBE) statement: guidelines for reporting observational studies. Lancet. 2007;370(9596):1453–7. 18064739 10.1016/S0140-6736(07)61602-X

[23] Lonardi S, et al. Analysis of early distant metastases of STAR-01: a randomized phase III trial comparing preoperative chemoradiation with or without oxaliplatin in locally advanced rectal cancer. American Society of Clinical Oncology; 2016. 10.1200/JCO.2010.34.4911 21606427

[24] Azria Daa, et al. Late toxicities and clinical outcome at 5 years of the ACCORD 12/0405-PRODIGE 02 trial comparing two neoadjuvant chemoradiotherapy regimens for intermediate-risk rectal cancer. Ann Oncol. 2017;28(10):2436–42. 28961836 10.1093/annonc/mdx351

[25] Yaghobi Joybari A, et al. Comparison of capecitabine (xeloda) vs. combination of capecitabine and oxaliplatin (XELOX) as neoadjuvant CRT for locally advanced rectal cancer. Pathol Oncol Res. 2019;25(4):1599–605. 30712194 10.1007/s12253-019-00587-3

[26] Cho MS, et al. Short-term outcomes and cost-effectiveness between long-course chemoradiation and short-course Radiotherapy for locally advanced rectal Cancer. Yonsei Med J. 2023;64(6):395. 37226566 10.3349/ymj.2023.0042 PMC10232996

[27] Haddad P, et al. Addition of oxaliplatin to neoadjuvant radiochemotherapy in MRI-defined T3, T4 or N + rectal cancer: a randomized clinical trial. Asia‐Pacific J Clin Oncol. 2017;13(6):416–22. 10.1111/ajco.12675 28488380

[28] Zhang H, et al. Impact of total neoadjuvant therapy consisting of consolidation chemotherapy on locally advanced rectal cancer survival. Int J Colorectal Dis. 2022;37(7):1657–68. 35716183 10.1007/s00384-022-04179-7

[29] Hoendervangers S, et al. Pathological complete response following different neoadjuvant treatment strategies for locally advanced rectal cancer: a systematic review and meta-analysis. Ann Surg Oncol. 2020;27:4319–36. 32524461 10.1245/s10434-020-08615-2 PMC7497700

[30] Gérard J-P, et al. Preoperative concurrent chemoradiotherapy in locally advanced rectal cancer with high-dose radiation and oxaliplatin-containing regimen: the Lyon R0–04 phase II trial. J Clin Oncol. 2003;21(6):1119–24. 12637479 10.1200/JCO.2003.10.045

[31] Greto D, et al. Neoadjuvant oxaliplatin and 5-fluorouracil with concurrent radiotherapy in patients with locally advanced rectal cancer: a single-institution experience. Radiol Med. 2013;118(4):570–82. 23358814 10.1007/s11547-012-0909-4

[32] Lee W-S, et al. Neoadjuvant treatment of mid-to-lower rectal cancer with oxaliplatin plus 5-fluorouracil and leucovorin in combination with radiotherapy: a Korean single center phase II study. Int J Clin Oncol. 2013;18:260–6. 22350021 10.1007/s10147-011-0372-6

[33] Chang H, et al. Optimize the dose of oxaliplatin for locally advanced rectal cancer treated with neoadjuvant chemoradiotherapy followed by radical surgery and adjuvant chemotherapy. BMC Cancer. 2020;20:1–10. 10.1186/s12885-020-06988-x PMC7268650 32487091

[34] Hong YS, et al. Oxaliplatin, fluorouracil, and leucovorin versus fluorouracil and leucovorin as adjuvant chemotherapy for locally advanced rectal cancer after preoperative chemoradiotherapy (ADORE): an open-label, multicentre, phase 2, randomised controlled trial. Lancet Oncol. 2014;15(11):1245–53. 25201358 10.1016/S1470-2045(14)70377-8

[35] Jiao D, et al. Fluorouracil-based preoperative chemoradiotherapy with or without oxaliplatin for stage II/III rectal cancer: a 3-year follow-up study. Chin J Cancer Res. 2015;27(6):588. 26752933 10.3978/j.issn.1000-9604.2015.12.05 PMC4697105

[36] Tang J, et al. Long-term outcome of oxaliplatin and capecitabine (XELOX) concomitant with neoadjuvant radiotherapy and extended to the resting period in high risk locally advanced rectal cancer. J Cancer. 2018;9(8):1365. 29721045 10.7150/jca.23874 PMC5929080

[37] Garcia-Aguilar J, et al. Preliminary results of the organ preservation of rectal adenocarcinoma (OPRA) trial. American Society of Clinical Oncology; 2020.

